# A systematic review of patient prioritization tools in non-emergency healthcare services

**DOI:** 10.1186/s13643-020-01482-8

**Published:** 2020-10-06

**Authors:** Julien Déry, Angel Ruiz, François Routhier, Valérie Bélanger, André Côté, Daoud Ait-Kadi, Marie-Pierre Gagnon, Simon Deslauriers, Ana Tereza Lopes Pecora, Eduardo Redondo, Anne-Sophie Allaire, Marie-Eve Lamontagne

**Affiliations:** 1 Department of Rehabilitation, Université Laval, Québec, Canada; 2 Centre interdisciplinaire de recherche en réadaptation et intégration sociale (CIRRIS), Centre intégré universitaire de santé et de services sociaux de la Capitale-Nationale, Québec, Canada; 3 Faculty of Business Administration, Université Laval, Québec, Canada; 4 Centre interuniversitaire de recherche sur les réseaux d’entreprise, la logistique et le transport (CIRRELT), Université de Montréal, Montréal, Canada; 5 Department of Logistics and Operations Management, HEC Montréal, Montréal, Canada; 6 Centre de recherche du CHU de Québec, Université Laval, Québec, Canada; 7 Centre de recherche en gestion des services de santé, Université Laval, Québec, Canada; 8 Department of Mechanical Engineering, Université Laval, Québec, Canada; 9 Faculty of Nursing, Université Laval, Québec, Canada

**Keywords:** Patient prioritization, Systematic review, Healthcare services, Waiting lists, Outcomes

## Abstract

**Background:**

Patient prioritization is a strategy used to manage access to healthcare services. Patient prioritization tools (PPT) contribute to supporting the prioritization decision process, and to its transparency and fairness. Patient prioritization tools can take various forms and are highly dependent on the particular context of application. Consequently, the sets of criteria change from one context to another, especially when used in non-emergency settings. This paper systematically synthesizes and analyzes the published evidence concerning the development and challenges related to the validation and implementation of PPTs in non-emergency settings.

**Methods:**

We conducted a systematic mixed studies review. We searched evidence in five databases to select articles based on eligibility criteria, and information of included articles was extracted using an extraction grid. The methodological quality of the studies was assessed by using the Mixed Methods Appraisal Tool. The article selection process, data extraction, and quality appraisal were performed by at least two reviewers independently.

**Results:**

We included 48 studies listing 34 different patient prioritization tools. Most of them are designed for managing access to elective surgeries in hospital settings. Two-thirds of the tools were investigated based on reliability or validity. Inconclusive results were found regarding the impact of PPTs on patient waiting times. Advantages associated with PPT use were found mostly in relationship to acceptability of the tools by clinicians and increased transparency and equity for patients.

**Conclusions:**

This review describes the development and validation processes of PPTs used in non-urgent healthcare settings. Despite the large number of PPTs studied, implementation into clinical practice seems to be an open challenge. Based on the findings of this review, recommendations are proposed to develop, validate, and implement such tools in clinical settings.

**Systematic review registration:**

PROSPERO CRD42018107205

## Background

Patients from countries with publicly funded healthcare systems frequently experience excessive wait times [1], with sometimes dramatic consequences. Patients waiting for non-urgent health services such as elective surgeries and rehabilitation services can suffer physical and psychological sequelae [2]. To reduce these negative effects, waiting lists should be managed as fairly as possible to ensure that patients with greater or more serious needs are given priority for treatment [3].

Patient prioritization, defined as the process of ranking referrals in a certain order based on criteria, is one of the possible strategies to improve fairness in waiting list management [4]. This practice is used in many settings, and it differs from a first-in first-out (FIFO) approach that ranks patients on waiting lists chronologically, according to their arrival. It also differs from triage methods used in emergency departments where patients are sorted into broader categories (e.g., low/moderate/high priority or service/no service). Prioritization is related to non-urgent services involving a broader range of timeframes and patient types [2, 5]. The use of prioritization is widely reported, yet not fully described, in services provided by allied health professionals, including physical therapists, occupational therapists, or psychologists as well as multidisciplinary allied health teams [2]. In practice, however, assessing patients’ priority on the basis of explicit criteria is complex and, to a certain degree, inconsistent [6–8]. Moreover, most prioritization criteria are subjectively defined, and comparing patients’ needs and referrals can be challenging [8].

Patient prioritization tools are designed to support the decision process leading to patient sorting in an explicit, transparent, and fair manner. Such tools or systems are usually set up to help clinicians or managers to make decisions about which patients should be seen first when demand is great and resources are limited [2]. We define patient prioritization tools as paper-and-pencil or computer-based instruments that support patient prioritization processes, either by stating explicit and standardized prioritization criteria, or by enabling easier or better calculation of priority scores, or because they automatically include the patients into a ranked list. PPTs are mainly built around sets of general criteria that encompass personal factors (i.e., age), social factors (i.e., ability to work), clinical factors (i.e., patients’ quality of life), and any other factor deemed relevant [1, 3, 5]. Since tools can take various forms and they are very dependent on the particular context of application, some of the literature reports a lack of consistency in the way they are developed [5, 6].

In healthcare, the process of validating a new tool to measure an abstract phenomenon such as quality of life, patient adherence, and urgency/needs in the case of PPTs, is in large part oriented toward the evaluation and the reduction of errors in the measurement process [9]. The process of a measure, a tool, or an instrument’s quality assessment involves investigating their reliability and validity. Reliability estimates are used to evaluate the stability of measures gathered at different time intervals on the same individuals (intra-rater reliability), the link and coherence of items from the same test (internal consistency), or the stability of scores for a behavior or event using the same instrument by different observers (inter-rater reliability) [9]. There are many approaches to measure validity of a tool and in the context of our review, we identified those more relevant. Construct validity is the extent to which the instrument measures what it says it measures [10]. Content validity addresses the representativeness of the measurement tool in capturing the major elements or different facets relevant to the construct being measured, which can be in part assessed by stakeholders’ opinion (face validity) [10]. Criterion-related validity evaluates how closely the results of a tool correspond to the results of another tool. As in the case of patient prioritizing, much of the research conducted in healthcare involves quantifying abstract concepts that cannot be measured precisely, such as the severity of a disease and patient satisfaction. Validity evidence is built over time, with validations occurring in a variety of populations [9].

Aside from the validation process, there is no consensus in the literature about the effect of PPTs on healthcare service delivery, patient flow, or stakeholders. Some studies state that the prioritization process is associated with lower waiting times [11–13], but a systematic review of triage systems indicates mixed results on waiting time reduction [14]. It is difficult to assess these findings considering the variance between research settings and the nature of the considered prioritization system, tool, or process used in the studies. For example, Harding et al. [14] included studies about any system that either ranked patients in order of priority or sorted patients into the most appropriate service, which are two completely different systems. These authors also merged results from emergency and non-emergency settings, which are contrasting contexts of healthcare. Emergency refers to contexts where patients present life-threatening symptoms requiring immediate clinical action. In the case of non-urgent healthcare services, also referred to as elective services, access is organized according to priorities and level of need [3]. Besides the outcome related to review evidence that highlights other indicators of quality related to PPTs and their effects on the process of care and users.

The prioritization process in various non-urgent settings may vary significantly according to the kind of PPT used. The heterogeneity of the tools is reflected in the array of outcomes used to evaluate the effectiveness of PPT, which makes it difficult to have a broader and systematic understanding of their real impact on clinical practices and patient health outcomes.

The goal of this paper is to systematically review and synthesize the published evidence concerning PPTs in non-emergency settings in order to (1) describe PPT characteristics, such as format, scoring description, population, setting, purpose, criteria, developers, and benefits/limitations, (2) identify the validation approaches proposed to enhance the quality of the tools in practice, and (3) describe their effect or outcome measures (e.g., shorter wait times).

## Methods

The detailed methods of this systematic review are reported in a published protocol [15], but some key elements are the following. The review has been registered in the PROPERO database (CRD42018107205). This review is based on the stages proposed by Pluye and Hong [16] to guide systematic reviews and it is reported in accordance with the Preferred Reporting Items for Systematic Reviews and Meta-Analyses (PRISMA) statement [17]. The PRISMA 2009 checklist is joined in Additional file 1.

### Search strategy

Two of the authors (JD and MEL), helped by a professional librarian, identified the search strategy for this systematic review in accordance with the research objectives. An example of a search query in the Medline (Ovid) database is presented in Additional file 2. First, JD and SD imported into a reference management software (Endnote) the records from five source databases: Medline, Embase, CINAHL, Web of Science, and the Cochrane Library. We did not apply any restrictions on the papers’ publication date. A secondary search was performed according to the following four steps: (1) screening of the lists of references in the articles identified; (2) citation searches performed using Google Scholar for records that meet the inclusion criteria; (3) screening of 25 similar references suggested by the databases, where available; and (4) contacting the researchers who authored two articles or more included in our review.

### Eligibility criteria

We selected articles that met the following inclusion criteria: (1) peer-reviewed quantitative/qualitative/mixed methods empirical studies, which includes all qualitative research methods (i.e., phenomenological, ethnographic, grounded theory, case study, and narrative) and quantitative research designs (i.e., randomized controlled studies, cohort studies, case control, cross sectional, descriptive, and longitudinal); (2) published in English or French; (3) reporting the use of a PPT in a non-emergency healthcare setting. We excluded references based on the following four exclusion criteria: (1) studies focusing on strategies/methods of waiting list management not using a prioritization tool or system; (2) studies conducted to fit an emergency setting; (3) articles dealing with critical or life-threatening situations (i.e., organ transplants); and (4) literature reviews.

### Screening and selection process

Two steps were applied to remove duplicates. First, we used the software command “Find duplicates” on the “title” field of the references. Then, JD and MEL independently screened all the references identified from the search and manually deleted remaining duplicates. In the screening process, first, titles and abstracts were analyzed to extract relevant articles. Whenever a mismatch on the relevance of an article arose between JD and MEL, the authors discussed the paper until a consensus was reached. Second, JD read the full texts of all the extracted articles to select those that were relevant based on our eligibility criteria. MEL, AR, and VB validated the article selection, and disagreements were resolved by discussion.

### Data extraction

All articles in the final list were reviewed by JD using an extraction grid. First, JD extracted information related to the studies, such as the authors, title, year of publication, country, population, purpose of the study, and setting. Second, JD documented the information about the tool used in the study, including format, description, developers, development process, criteria, reliability, validity, and outcome assessment, relevant results, and implementation process. The grid and information extracted by JD were validated independently by six research team members.

### Data synthesis

We used a data-based convergent qualitative synthesis method to describe the results of the systematic mixed studies review [16]. As described by Hong et al. [18], all included studies are analyzed using the same synthesis method and results are presented together. Since only one synthesis method is used for all evidence, data transformation is involved. In our review, results from all studies were transformed into qualitative findings [18]. We extracted qualitative data related to the objectives of the review from all the manuscripts included. In the extraction grid, we conducted a hybrid deductive-inductive approach [18] using predefined themes (e.g., developers and prioritization criteria), then we assigned data to themes, and new themes derived from data (e.g., types of outcome measured in the included studies). Quantitative data presented in our review are the numbers of occurrences of the qualitative data extracted in the included studies.

### Critical appraisal

The methodological quality of the studies selected was assessed by three independent assessors (JD, ATP, ASA) using the mixed methods appraisal tool (MMAT-version 2018) [19, 20], which, on its own, allows for concomitantly appraising all types of empirical studies, whether mixed, quantitative, or qualitative [19]. Each study was assigned a score, from 0 to 5, based on the number of criteria met. We did not exclude studies with low MMAT scores.

## Results

### Description of included articles

**Fig. 1:**
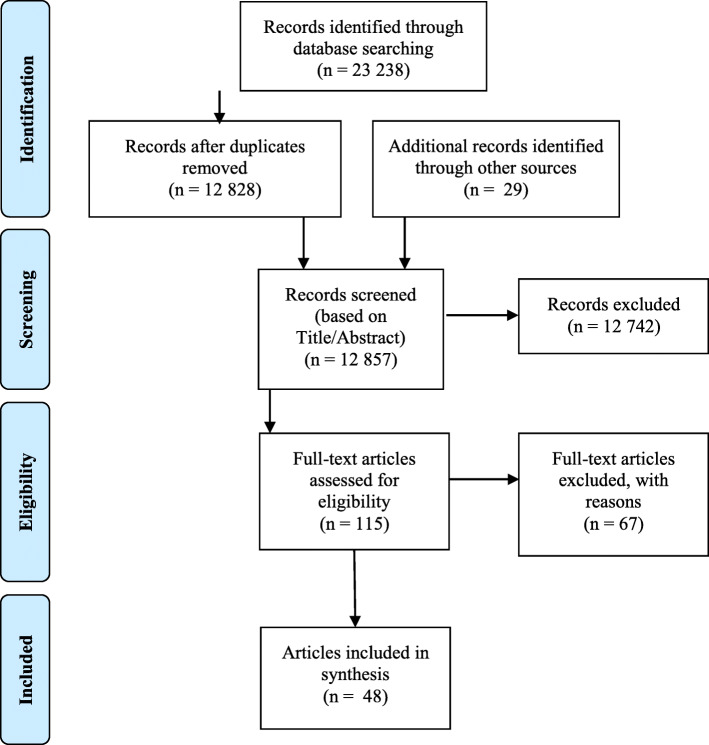
PRISMA flow diagram from Moher et al. [17]

The database search was conducted from 24 September 2018, to 14 January 2019. It was updated on 4 November 2019. We screened a total of 12,828 records after removing duplicates. We assessed 115 full-text articles for eligibility and from those, 67 were excluded based on at least one exclusion criterion. Figure 1 presents the PRISMA flow diagram for inclusion of the 48 relevant papers [13, 21–67].

The articles included were published between 1989 and 2019. Most of the studies were conducted in Canada [22, 23, 25, 26, 30–33, 40, 42, 48, 50, 53, 57, 59, 61, 67], Spain [21, 28, 29, 37, 38, 41, 52, 54–56, 60, 62], and New Zealand [27, 34–36, 49, 58, 63]. As presented in Table 1, these studies’ stated goals were mainly related to evaluating validity and reliability, developing prioritization criteria, and creating prioritization tools.

**Table 1:** Study characteristics of the included articles related to each PPT

Healthcare service	Number of PPTs	(#ref) Authors (year)	Country	Purpose of the studies	Healthcare setting	Quality rating^**a**^
Arthroplasty	9	[21] Allepuz et al. (2008)	Spain	Evaluate validity and reliability of the PPT	Hospital	4
		[29] Comas et al. (2010)		Measure outcomes of the PPT, including waiting times		5
		[62] Tebe et al. (2015)		Simulate models to compare PPT to FIFO		4
		[38] Escobar et al. (2007)	Spain	Develop a PPT	Hospital	4
		[37] Escobar et al. (2009)		Evaluate the validity of the PPT		5
		[55] Quintana et al. (2000)	Spain	Evaluate reliability and validity of the PPT	Hospital	4
		[22] Arnett et al. (2003)	Canada	Develop prioritization criteria and evaluate reliability	Hospital	4
		[30] Conner-Spady et al. (2004)		Evaluate the validity of the PPT		4
		[31] Conner-Spady et al. (2004)		Evaluate validity and reliability of the PPT		3
		[33] De Coster et al. (2007)	Canada	Develop a PPT	Primary care	5
		[53] Naylor and Williams (1996)	Canada	Develop prioritization criteria	Hospital	4
		[67] Rahimi et al. (2016)	Canada and Iran	Develop prioritization criteria and simulate models to compare to FIFO	Hospital	2
		[27] Coleman et al. (2005)	New Zealand	Evaluate the validity of the PPT	Outpatient clinic	3
		[63] Theis (2004)	New Zealand	Evaluate the validity of the PPT	Hospital	3
Cataract surgery	5	[21] Allepuz et al. (2008)	Spain	Evaluate validity and reliability of the PPT	Hospital	4
		[28] Comas et al. (2008)		Simulate models and compare it with FIFO		5
		[56] Quintana et al. (2006)	Spain	Develop prioritization criteria	Hospital	3
		[41] Gutiérrez et al. (2009)		Evaluate the validity of the PPT		5
		[57] Romanchuk et al. (2002)	Canada	Develop prioritization criteria and evaluate their reliability	Hospital	3
		[32] Conner-Spady et al. (2005)		Evaluate the validity of the PPT		5
		[47] Lundström et al. (2006)	Sweden	Develop a PPT and evaluate its validity and reliability	Outpatient clinic	4
		[13] Ng and Lundstrom (2014)		Evaluate waiting times using a PPT		3
		[39] Fantini et al. (2004)	Italy	Develop prioritization criteria	Unknown	4
Other elective surgery	4	[34] Dennett and Parry (1998)	New Zealand	Evaluate the validity of the PPT	Hospital	4
		[35] Derrett et al. (2003)		Evaluate the validity of the PPT		5
		[49] McLeod et al. (2004)		Understand the use of, and attitudes to the PPT		5
		[36] Dew et al. (2005)		Explore processes and progress of PPT implementation		5
		[60] Solans-Domènech et al. (2013)	Spain	Develop a PPT	Hospital	5
		[61] Taylor et al. (2002)	Canada	Develop prioritization criteria and evaluate their reliability	Hospital	4
		[64] Valente et al. (2009)	Italy	Evaluate waiting list management on the basis of clinical urgency and waiting time	Unknown	5
Orthodontic treatment	2	[24] Brook and Shaw (1989)	United Kingdom	Develop a PPT	Hospital	3
		[51] Mohlin and Kurol (2003)	Sweden	Examine whether PPT are sensitive enough to select cases in mid-range treatment needs	Unknown	1
Psychiatry	2	[44] Isojoki et al. (2008)	Finland	Explore whether the PPT score is associated with the treatment received	Outpatient clinics	4
		[45] Kaukonen et al. (2010)	Finland	Develop and validate criteria of the PPT	Hospital (inpatient and outpatient)	4
Mental health (youth and adult)	2	[59] Smith et al. (2002)	Canada	Develop a PPT and evaluate its reliability	Hospital and community-based care	4
		[26] Cawthorpe et al. (2007)		Evaluate the validity of the PPT		5
		[23] Boucher (2016)	Canada	Develop a PPT and adapt it to a different population	Primary care	3
Bariatric surgery	1	[54] Pérez et al. (2018)	Spain	Develop a PPT	Hospital	2
Chronic care	1	[25] Burkell et al. (1996)	Canada	Simulate and compare models with FIFO	Hospital	3
Coronary artery bypass surgery	1	[58] Seddon et al. (1999)	New Zealand	Review the PPT score and determine whether it prioritizes patients at high risk of cardiac events while waiting	Hospital	4
MRI	1	[42] Hadorn et al. (2002)	Canada	Develop a PPT, criteria, and evaluate their reliability	Unknown	3
Occupational therapy	1	[43] Heasman and Morley (2012)	United Kingdom	Develop prioritization criteria and a PPT	Hospital, community-based care, and rehabilitation	5
Physiotherapy	1	[50] Mifflin and Bzdell (2010)	Canada	Develop a PPT	Rehabilitation	3
Psychotherapeutic service	1	[65] Walton and Grenyer (2002)	Australia	Evaluate the reliability and validity of the PPT	Community-based care	5
Rheumatology	1	[40] Fitzgerald et al. (2011)	Canada	Develop a PPT and evaluate its reliability	Primary care	3
Varicose vein surgery	1	[52] Montoya et al. (2014)	Spain	Develop a PPT and evaluate its validity and reliability	Hospital, outpatient clinics, and primary care	5
Elective admission	1	[66] Zhu et al. (2019)	China	Develop prioritization criteria and a PPT	Hospital	5

^a^Quality score ranged from meeting none of five criteria (0) to meeting all criteria (5)

Three development processes of similar tools stand out in our review based on the number of studies conducted, one from the Western Canada Waiting List Project [68], which produced 12 studies included in our review [22, 26, 30–33, 40, 42, 48, 57, 59, 61], one from Spanish research groups including 10 studies [21, 28, 29, 37, 38, 41, 55, 56, 60, 62], and one from four New Zealand researcher studies [34–36, 49]. They are reviewed in more detail in the following paragraphs.

### Western Canada Waiting List (WCWL) project

The WCWL project was a collaborative initiative undertaken by 19 partner organizations to address five areas where waiting lists were considered to be problematic: cataract surgery, general surgery, hip and knee replacement, magnetic resonance imaging (MRI), and children’s mental health services [68]. Hadorn’s team developed point-count systems using statistical linear models [68]. These types of systems have been developed for many clinical and non-clinical contexts such as predicting mortality in intensive care units (APACHE scoring system [69]) and neonatal assessment (Apgar score [70]). In this project, the researchers developed tools using priority scores in the 0-100 range based on weighted prioritization criteria. Based on the studies included in our review, a panel of experts adopted a set of criteria, incorporated them in a questionnaire to rate a series of consecutive patients in their practices, and then used regression analysis to determine the statistically optimal set of weights on each criterion to best predict (or correlate with) overall urgency [42, 57, 59, 61]. Reliability [22, 33, 40, 42, 57, 61] and validity [26, 30–33] were also assessed for most of the tools created by this research team and the key results are presented in Additional file 4.

### Spain

A group of researchers in Spain developed a four-step approach to designing prioritization tools. The four steps included (1) systematic review to gather available evidence about waiting list problems and prioritization criteria used, (2) compilation of clinical scenarios, (3) expert panel consultations provided with the literature review and the list of scenarios, (4) rating of the scenarios and criteria weighting, carried out in two rounds using a modified Delphi method. The researchers then evaluated the reliability of the tool in the context of hip and knee surgeries [21, 38, 55] as well as its validity for cataract surgeries [21, 37, 41, 56] and these results are detailed in Additional file 4.

### New Zealand

The third tool detailed in our review was developed by New Zealand researchers. Clinical priority assessment criteria (CPAC) were defined for multiple elective surgery settings [35, 36, 49] based on a previous similar work [34]. Validity results of these tools are presented in Additional file 4. These tools were part of an appointment system aimed at reforming the access to elective surgery policy in order to improve equity, provide clarity for patients, and achieve a paradigm shift by relating likely benefits from surgery to the available resources [36]. The development process was not described in detail in the studies included. As discussed in one study, implementation of the system encountered some difficulties, mostly in achieving consensus on the components and the weighting of the various categories of prioritization criteria [36].

### Designs and quality of included studies

We appraised the methodological quality of the 48 articles using MMAT, which allowed for quality appraisal based on the design of the studies assessed. One was a mixed methods study [50], four were qualitative studies [36, 43, 48, 60], five were quantitative descriptive studies [35, 37, 49, 52, 66], none were randomized controlled trials, and the remaining 38 were quantitative non-randomized studies. From these quantitative studies, most were cross sectional, prospective, or retrospective design studies. The overall methodological quality of the articles was good, with a mean score of 3.81/5 (range from 0 to 5). Score associated with each study is presented in Table 1.

### Characteristics of the prioritization tools

We listed 34 distinct PPTs from 46 articles reviewed. Two articles [46, 48] were included even though no specific PPT was used or developed in these studies. In Kingston and Masterson’s study [46] (MMAT score = 0), the Harris Hip Score and the American Knee Society Score were used as the scoring instruments to determine priority in the waiting list, and in McGurran et al.’s article [48], the (MMAT score = 2) authors consulted the general public to collect their opinion on appropriateness, acceptability, and implementation of waiting list PPTs. Table 1 shows that PPTs were mostly used in hospital settings (19/34) for arthroplasty (9/34), cataract surgery (5/34), and other elective surgeries (4/34). We found that a different set of tools support prioritization in 14 other healthcare services. Three tools were designed for primary care, three for outpatient clinics, one for community-based care, and one for rehabilitation. Three studies [26, 43, 52] portrayed the use of a PPT in multiple settings, and four studies [39, 42, 51, 64] did not specify the setting. The PPTs reviewed were mostly (17/34) tools attributing scores ranging from 0 to 100 to patients based on weighted criteria. Other tools (8/34) used priority scores that sorted patients into broad categories (e.g., low, intermediate, high priority). The format of the PPTs reported in the studies were mostly unspecified (26/34), but some explicitly specified that the tool was either in paper (4/34) or electronic (2/34) format.

**Fig. 2:**
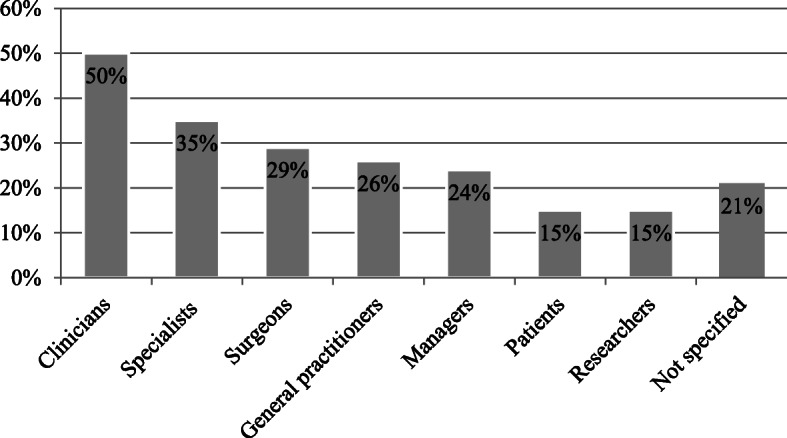
Stakeholders participating in the PPTs development

Several stakeholders were involved in the development of the 34 PPT retrieved, such as clinicians (50% of the PPT), specialists (35%), surgeons (29%), general practitioners (26%), and others (Fig. 2). It is worth mentioning that patients and caregivers were involved in only 15% of the PPTs developed [21, 38, 52, 60, 63, 65], while for 21% of the PPTs, authors did not specify who participated in their development.

Regarding the development process, we have not identified guidelines or standardized procedures explaining how the proposed PPTs were created. Some authors reported relying on literature reviews (44%) and stakeholder consultations (53%) to inform PPT design, but most provided very little information about the other steps of development.

Below is a review of the criteria elected to produce the different PPTs found in this synthesis. First, the number of criteria ranges from 2 to 17 (mean: 7.6, SD: 3.8). As regards their nature or orientation, some PPTs are related to generic criteria, others are specific to a disease, a service, or a population, as reported in Table 2. The criteria of each PPT are listed in Additional file 3.

**Table 2:** Type of criteria reported at least once in PPTs

	Number of PPT (/34)
Generic criteria	
Threat to the patient’s ability to play a role/independence	20
Functional limitations	19
Pain/suffering	15
Probability of recovery/progression of disease/prognosis	14
Advantages/benefits of intervention	10
Having or being a caregiver	7
Age	4
Level of urgency	3
Time on the waiting list	2
Specific criteria	
Related to symptoms	10
Related to other standardized measures	8

In summary, PPTs are typically used in hospital settings for managing access to hip, knee, cataract, and other elective surgeries. Their format is undefined, their development process is non-standardized, and they are mostly developed by consulting clinicians and physicians (surgeons, general practitioners, and specialists).

### Reliability and validity of PPTs

**Fig. 3:**
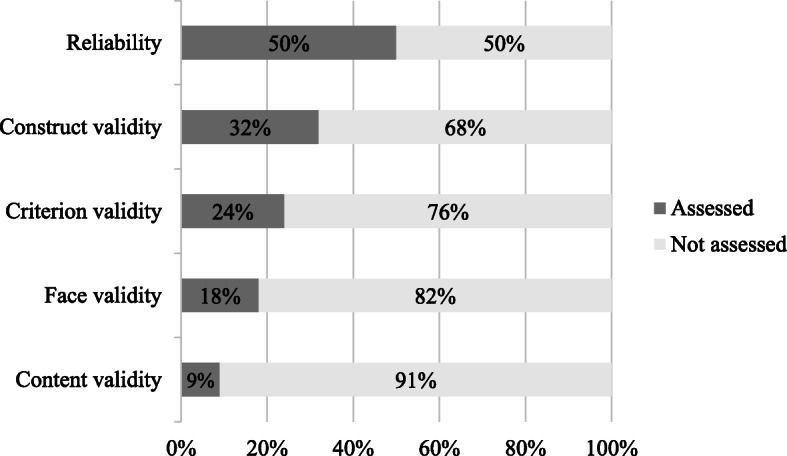
Portion of the 34 PPTs assessed for each aspect of quality

Only 26 out of the 48 articles included in this synthesis, representing 23 tools (68%), reported an investigation of at least one of the qualities of the measuring instrument, i.e., reliability and validity. Figure 3 displays the scope of aspects that were assessed.

Inter-rater [21–24, 33, 38, 40, 42, 45, 47, 50, 55, 57, 61, 65] and intra-rater [22, 24, 33, 40, 42, 47, 55, 57, 61] reliability were evaluated by comparing the priority ratings of two groups of raters (inter) and by comparing priority ratings by the same raters at two different points in time (intra). Face validity [33, 38, 41, 47, 52, 55] was determined by consultation with stakeholders (e.g., surgeons, clinicians, patients, etc.). Other validity assessments, such as content [41, 47, 55], construct [21, 23, 26, 27, 30–32, 34, 35, 37, 52, 63], and criterion [26, 32, 35, 41, 45, 47, 52, 55] validity were appraised using correlations between PPT results and other measures. In fact, some studies compared PPT scores with a generic health-related quality of life measure such as the Short Form Health Survey (both SF-36 and SF-12) [35, 55, 63]. Aside from correlations with other measures, PPT validity was evaluated using two other means: disease-specific questionnaires (e.g., the Western Ontario and McMaster Universities Arthritis Index [27, 30, 31, 37, 55] and the Visual Function Index [35, 41]) or another measure of urgency/priority (e.g., the Visual Analog Scale [21, 22, 26, 30–32, 42, 61] or a traditional method [47]).

One of the objectives of this review was to synthesize results about the quality features of each PPT. The diversity of contexts, settings, and formats PPTs adopted made a fair and reasonable comparison almost impossible. We observed various methods of assessing reliability and validity of PPTs across a number of settings. All the findings relating to the features reported in the articles are presented in Additional file 4. We can conclude that the reliability and the validity of PPTs have generally been assessed as acceptable to good.

### Effects on the waiting list process

Assessing actual benefits remains, in our opinion, one of the most important drawbacks of the reported PPTs. In fact, we found that only 10 studies [13, 28, 29, 39, 44, 46, 50, 58, 62, 64] investigated the effects or outcomes of the proposed PPTs, while six other studies [23, 36, 48–50, 67] merely reported opinions expressed by stakeholders about essential benefits and limitations of PPTs.

Waiting time is the most studied outcome assessment [13, 28, 29, 39, 58, 62, 64]. Four studies [28, 29, 39, 62] used a computer simulation model to evaluate the impact of the PPT on waiting times. In their simulations, the authors compared the use of a PPT to the FIFO model and reported mixed findings. Comas et al. [28, 29] showed that prioritization systems produced better results than a FIFO strategy in the contexts of cataract and knee surgeries. They concluded that the waiting times weighted by patient priority produced by prioritization systems were 1.54 and 4.5 months shorter than the ones produced by FIFO in the case of cataract and knee surgeries, respectively. Another study [39] revealed, in regard to cataract surgery, that the prioritization system concerned made it possible for patients with the highest priority score (91-100) to wait 52.9 days less than if the FIFO strategy were used. In contrast, patients with the lowest priority score (1-10) saw their mean waiting time increase from 193.3 days (FIFO) to 303.6 days [39]. Tebé et al. [62] noted that the application of a system of prioritization seeks to reorder the list so that patients with a higher priority are operated on earlier. However, this does not necessarily mean an overall reduction in waiting times. These authors concluded that although waiting times for knee arthroplasty dropped to an average wait of between 3 and 4 months throughout the period studied, they could not ascertain that it was directly related to the use of the prioritization system [62].

The other three studies conducted a retrospective analysis of patients on waiting lists [13, 58, 64]. With a PPT [13] using a total score of priority then sorting patients in groups (group 1 having the greatest need for surgery and group 4 the least need), the mean waiting time for surgery was 3 years shorter across all indication groups. In a study with patients waiting for cardiac surgery, the clinician’s classification was compared to the New Zealand priority scores (0-100) based on clinical features [58]. According to this study, it is difficult to determine whether waiting times were reduced as a result of the use of the PPT, because findings only showed the reorganization of the waiting list based on each category and priority scores. However, waiting times were reduced for the least urgent patients in both groups. Waiting times before surgery were between 161 and 1199 days based on the clinician’s classifications compared to waiting times related to New Zealand priority scores, which were between 58 and 486 days [58]. In addition, Valente et al. [6] studied a model to prioritize access to elective surgery and found no evident effects in terms of reduction or increase of the overall waiting list length.

### Effects on the care process

Some studies address the effects of PPT on the demand for healthcare services [44, 46, 50, 64]. The introduction of a new need-based prioritization system for hip and knee arthroplasty has reduced the number of inquiries and cancelations [46]. Changes were also observed after the implementation of a PPT for physiotherapy services with an increase of 38% of high priority clients in their caseload [50]. PPTs also had an impact on the healthcare delivery process. For example, Mifflin and Bzdell [50] reported improvement in communication between physiotherapists and other health professionals in remote areas. Furthermore, Isojoki et al. [44] demonstrated that the priority ratings made by experts in adolescent psychiatry were correlated with the type and duration of the treatment received. This suggests that the PPT identified adolescents with the greatest need of psychiatric care and that it might, to some extent, predict the intensity of the treatment to be delivered [44]. The system proposed by Valente et al. [64] enabled easy and coherent scheduling and reduced postponements [64].

### Other outcomes related to the use of PPTs

Although some attempts have been made to assess the positive impacts of PPTs on patients, the results reported are not consistent enough to confirm such benefits. Seddon et al. [58] stated that priority scores for cardiac surgery prioritize patients as accurately as clinician assessments do according to the patients’ risk of cardiac events (cardiac death, myocardial infarction, and cardiac readmission). In their study concerning a PPT for patients waiting for hip and knee arthroplasty, Kingston and Masterson [46] used two instruments to measure patients’ priority scores (the Harris Hip Score and the American Knee Society Score), and the mean joint score for patients on the waiting list remained unchanged a year after the introduction of the new system.

### Benefits and limitations of PPTs

The challenge of producing long-term assessments of PPT benefits has led researchers to rely on qualitative methods, i.e., stakeholder perceptions concerning acceptability and benefits of PPTs. Focus groups including the general public indicated that the tools presented in five different clinical areas^1^ were appropriate and acceptable [48]. Other studies [23, 36, 49, 50, 67] examined perceptions of PPT stakeholders—clinicians, managers, and surgeons—about the tools. Clinicians using a PPT in clinical practice stated that it promotes a shared and more homogeneous vision of patients’ needs and that it helps to gather relevant information about them [23]. It also improved transparency and equity for patients, as well as accuracy of waiting times [36]. In another study, physiotherapists reported increased job satisfaction, decreased job stress, and less time spent triaging referrals [50]. They also commented that, compared to the methods used before the tool was introduced [50], PPT allowed physiotherapy services to be delivered more equitably. In Rahimi et al.’s study [67], surgeons reported that the PPT provides a precise and reliable prioritization that is more effective than the prioritization method currently in use.

On a less positive note, some authors reported that PPTs were perceived as lacking flexibility, which limited their acceptance by surgeons [36, 49]. In a study surveying surgeons about the use of PPTs, only 19.5% agreed that current PPTs were an effective method of prioritizing patients, and 44.8% felt that further development of surgical scoring tools had the potential to provide an effective way of prioritizing patients [49]. In fact, most surgeons felt that their clinical judgment was the most effective way of prioritizing patients [49]. Many studies mentioned the need to support implementation of PPTs in clinical practice and to involve potential tool users in the implementation process [21, 31, 48, 57, 60]. In this vein, another recommendation concerning implementation was to secure agreement and to assess acceptability of the criteria and the tool in clinical settings [60]. A panel of experts recommended that a set of operational definitions and instructions be prepared to accompany the criteria in order to make the tool more reliable [57]. Implementation should also involve continuous monitoring and an evaluation of the effects of implementation on patient outcomes, on resource use, and on the patient-provider relationship [31].

## Discussion

The aim of this systematic review was to gather and synthesize information about PPTs in non-emergency healthcare contexts in order to develop a better understanding of their characteristics and to find out to what extent they have been proven to be reliable, valid, and effective methods of managing access to non-urgent healthcare services. A significant number of PPTs are discussed in the literature, most of them designed to prioritize patients waiting for elective surgeries such as, but not limited to, cataract, knee, and hip operations. We also discovered a broad range of studies assessing PPT reliability and validity. The wide range of methods underlying the tools and contexts in which they are used to make a fair assessment of their quality very difficult. We nonetheless noted that the overall assessment of PPT reliability and validity were reported as being acceptable to good. We have also synthesized the quality assessment processes conducted in the literature to apprise interested readers and encourage further research on PPT development and validation. The overall effectiveness of PPTs was difficult to demonstrate according to our findings, essentially because the articles showed mixed results about reduction of waiting times and because of the lack of studies measuring PPT impact on patients. However, a few studies demonstrated benefits according to the general public, clinicians, and physicians, in terms of a more equitable and reliable delivery of services. PPTs are used to manage access to a given healthcare service by helping service providers to prioritize patients on a waiting list. The use of PPTs as scoring measures provides a relatively transparent and standardized method for assigning priority to patients on waiting lists [68]. Measuring abstract constructs, such as the relative priority of the patients’ need to obtain access to a healthcare service is, indeed, a complex task.

The findings of this review show that PPT development processes are, at best, heterogeneous. However, our systematic review demonstrates that certain development steps are present in most studies. Based on these results, and supported by studies that showed reliable and valid PPTs, we can draw some recommendations or guidelines for the development of reliable, valid, user-friendly, and acceptable tools (see Table 3). Recommendations are formulated regarding preliminary steps, development, evaluation, and finally the implementation of PPTs.

**Table 3:** Recommendations and guidelines for PPT development projects

Prior to tool development	Tool development	Evaluation and Implementation
1. Conducting a review of the literature to explore the prioritization tools that already exist for a given healthcare service. 2. Consulting multiple stakeholders (patients, clinicians, managers, researchers, etc.) to identify the need for prioritization tools.	1. Conducting a systematic review of the literature to identify evidence related to prioritization criteria. 2. Holding group consultations (e.g., nominal groups, Delphi, focus groups) to reach consensus about prioritization criteria to be included in the tool. 3. Involving various stakeholders in the consultations, such as patients, caregivers, and lay people, in order to have multiple points of view about the criteria. 4. Adapting the tool to the context, the setting, and the users (simple, precise, accessible, quick, etc.)	1. Testing the reliability (intra-raters and inter-raters) of the tool with a small sample of patients. 2. Assessing the validity of the tool by: a) consulting potential users to determine whether the tool is perceived as covering the concept it purports to measure. b) comparing to another priority measure (generic or specific to the given service). 3. Evaluating effects on processes and stakeholders. 4. Involving and supporting tool users during implementation.

Our systematic review broadens earlier research reviewing priority scoring tools used for prioritizing patients in elective surgery settings [5] to more general patient prioritization tools and to all non-urgent healthcare settings.

Harding et al. conducted two systematic reviews, which focused specifically on triage methods [6] and their impact on patient flow in a broad spectrum of health services [14]. Our review distinguishes prioritization tools and triage systems. Triage and prioritization are often used interchangeably because both terms refer to allocating services to patients at the point of service delivery [2]. In fact, triage was traditionally associated with emergency services, but it was also used in other healthcare settings, and it relates to the process of making a decision about the type of service needed (for example, the need for a physical therapist versus a therapy assistant) and whether there is such a need at all [2]. Prioritization is a process of ranking patient needs for non-urgent services and as such, it involves a broader range of timeframes and patient types [2, 5]. Whereas triage sorts patients into broader categories (e.g., low/moderate/high priority or service/no service), it can nevertheless imply a prioritization process, as presented in our results.

Harding et al.’s review [14] included any system that either ranked patients in order of priority, or sorted patients into the most appropriate service. Additionally, 64% of the studies included were conducted in acute care hospital emergency departments [14]. Since both terms coexist in the literature, we included studies that used prioritization tools, which only rank patients in order of priority. Based on this, we obtained a better picture of the prioritization tools used, and we found that prioritization is more often used in non-urgent healthcare settings, without using a sorting (triage) process.

By excluding studies in emergency and life-threatening settings, we can assume that the evidence reviewed relates more to an assessment of patient priority based on needs and social characteristics, and less to an urgency to receive a given service [3]. As demonstrated by the wide range of PPTs reviewed, prioritization is used in managing access to care across many healthcare settings other than emergency departments, such as elective surgeries, rehabilitation, and mental health services.

We listed 16 specific types of non-urgent healthcare services and we found that most of the criteria used are generic, such as the threat to the patient’s ability to play a role, functional limitations, pain, and probability of recovery. Two PPTs used only specific criteria related to the disease, i.e., varicose vein surgery [52] and orthodontic treatment [51]. We demonstrated that even prioritization tools used in specific health condition services included generic criteria to prioritize patients on waiting lists. These findings suggest that generic criteria, such as non-clinical or social factors, could be added to condition-specific criteria in PPTs to represent more fairly and precisely patients’ needs to receive healthcare services.

One of the limitations of this systematic review is that the search strategy was restricted to the English and French languages. The definition of PPT is neither precise nor systematic in the literature as some authors use other terms such as triage systems and priority scoring tools. This explains why our database search yielded a substantial number of references. However, we believe that our search strategy was rigorously conducted in order to include all relevant studies. Heterogeneity between healthcare services and settings in our review highlighted the wide variety of PPT uses, but it could limit the generalizability of the results. Considering the large number of studies and PPTs found in the literature, we presented our synthesis descriptively in order to define the current state of knowledge, but we did not intend to compare the tools reviewed to one another. Although we found that PPTs are broadly used in non-urgent healthcare services, we did not find any evidence about the prevalence of PPT use in current practice. We are aware that other PPTs may exist in very specific healthcare organizations, and that a grey literature search would surely benefit this review.

## Conclusion

Long waiting times and other problems of access to healthcare services are important challenges that public healthcare systems face. Patient prioritization could help to manage access to care in an equitable manner. Development and validation processes are widely described for PPTs in non-urgent healthcare settings, mainly in the contexts of elective surgeries, but implementation into clinical practice seems to be a challenge. Although we were able to put forward some recommendations to support the development of reliable and valid PPTs for non-urgent services, we believe that more standardized projects need to be conducted and supported in order to evaluate facilitators and barriers to the implementation of such innovations. Further research is also needed to explore the outcomes of PPT use—other than their effects on waiting times—in clinical settings.

## Supplementary information

**Additional file 1.** PRISMA 2009 Checklist.

**Additional file 2.** Example of search strategy in MEDLINE/Ovid database.

**Additional file 3.** Table describing all criteria included in PPTs.

**Additional file 4.** Key results regarding reliability and validity of PPTs.

## Data Availability

All data generated or analyzed during this study are included in this published article and its supplementary information files.

## Abbreviations

CINAHL: Cumulative Index to Nursing and Allied Health Literature
CPAC: Clinical priority assessment criteria
FIFO: First-in first-out
MMAT: Mixed methods appraisal tool
MRI: Magnetic resonance imaging
PPT: Patient prioritization tool
PRISMA: Preferred Reporting Items for Systematic Reviews and Meta-Analyses
SD: Standard deviation
WCWL: Western Canada Waiting List

## Footnotes

1 Hip and knee joint replacement; cataract removal surgery; general surgery; children’s mental health services; and MRI scanning.
