# Supplementary material for: A systematic review of patient prioritization tools in non-emergency healthcare services
Source: Syst Rev. 2020 Oct 6;9:227. doi: 10.1186/s13643-020-01482-8 (PMC7541289; doi:10.1186/s13643-020-01482-8)
Supplement: Supplementary file 2 — Additional file 2. Example of search strategy in MEDLINE/Ovid database. [file 13643_2020_1482_MOESM2_ESM.docx]

## Additional file 2

## Example of search strategy in MEDLINE/Ovid database

| **#** | **Search** |
| --- | --- |
| 1 | (client* or patient* or "service user*").ti. or (client* or patient* or "service user*").ab. |
| 2 | Patient Selection.sh. |
| **3** | **1 or 2** |
| 4 | (categor* or setting or strateg* or system* or tool*).ti. or (categor* or setting or strateg* or system* or tool*).ab. |
| 5 | Classification/ |
| **6** | **4 or 5** |
| 7 | (queu* or delay* or "waiting time*" or "wait time*" or "waiting list*" or "wait list*" or waitlist*).ti. or (queu* or delay* or "waiting time*" or "wait time*" or "waiting list*" or "wait list*" or waitlist*).ab. |
| 8 | Systems Theory.sh. |
| 9 | Waiting Lists.sh. |
| **10** | **7 or 8 or 9** |
| 11 | (priorit* or triag*).ti. or (priorit* or triag*).ab. |
| 12 | Health Priorities.sh. |
| 13 | Triage.sh. |
| **14** | **11 or 12 or 13** |
| 15 | ("health service*" or "healthcare service*").ti. or ("health service*" or "healthcare service*").ab. |
| 16 | Health Services.sh. |
| **17** | **15 or 16** |
| **18** | **3 or 6 or 10** |
| **19** | **14 and 18** |
| **20** | **17 and 19** |
