# Supplementary material for: A systematic review of patient prioritization tools in non-emergency healthcare services
Source: Syst Rev. 2020 Oct 6;9:227. doi: 10.1186/s13643-020-01482-8 (PMC7541289; doi:10.1186/s13643-020-01482-8)
Supplement: Supplementary file 3 — Additional file 3. Table describing all criteria included in PPTs. [file 13643_2020_1482_MOESM3_ESM.docx]

## Additional file 3

## Table describing all criteria included in PPTs

| **Healthcare service** | **Reference** | **Criteria** |
| --- | --- | --- |
| **Arthroplasty** | Allepuz 2008  Comas 2010  Tebe 2015 | 1. Severity of the disease 2. Pain 3. Probability of recovery 4. Difficulty in doing ADL 5. Limitation on ability to work, 6. Has someone to look after the patient, 7. Be a caregiver |
|  | Escobar 2007, 2009 | 1. Pain on motion 2. Walking functional limitations 3. Abnormal findings on physical examination 4. Pain at rest (13) 5. Social role 6. Other pathologies 7. Other functional limitations |
|  | Quintana 2000 | 1. Previous nonsurgical procedures 2. Pain level 3. Functional limitations 4. Age 5. Bone quality 6. Surgical risk |
|  | Arnett 2003  Conner-Spady 2004  Conner-Spady 2004 | 1. Pain on motion 2. Pain at rest 3. Ability to walk without significant pain 4. Other functional limitations 5. Abnormal findings on physical examination related to affected joint 6. Potential for progression of disease documented by radiographic findings 7. Threat to patient role and independence in society. |
|  | De Coster 2007 | 1. Pain on motion 2. Pain at rest 3. Ability to walk without significant pain 4. Other functional limitations 5. Abnormal findings on physical exam related to most severely affected joint 6. Highest level of medication to manage affected joint 7. Threat to patient role and independence in society 8. Urgency level |
|  | Naylor 1996 | 1. Rest pain 2. Pain on activities of daily living 3. Problem in work or care giving 4. Improvement in functional status expected |
|  | Rahimi 2016 | 1. Disease severity (pains, difficulty doing activities) 2. Age of patient 3. Responsibility of caring someone else 4. Effectiveness of surgery (becoming independent, probability-degree of improvement) 5. Social status of patient 6. Present comorbidity 7. Interest to surgery (surgeon, patient) 8. Having previous complicated surgery or revision surgery. 9. Risk of death 10. Risk of worsening severity 11. Risk of reduction on the surgery's effectiveness 12. Risk of complications, comorbidity, affecting adjacent organs or spread of the disease. |
|  | Coleman 2005 | 1. Patient’s need based on pain 2. Functional activity 3. Movement and deformity 4. Multiple joint involvement 5. Ability to care for self and dependents |
|  | Theis 2004 | 1. Natural history of condition 2. Pain 3. Functional limitation 4. Social limitation 5. Benefit from surgery |
| **Cataract surgery** | Allepuz 2008  Comas 2008 | 1. Visual impairment 2. Recovery probability 3. Difficulty in performing activities of daily living 4. Ability to work 5. Have someone to look after the patient 6. Be a caregiver |
|  | Quintana 2006  Gutierrez 2009 | 1. Appropriateness 2. Presence of ocular comorbidities 3. Pre-intervention visual acuity in the cataractous eye 4. Patient referred visual functioning pre-intervention 5. Visual acuity in the contralateral eye 6. Expected visual acuity following the intervention 7. Type of cataract (laterality) 8. Social dependency (need for support by others for activities of daily living). |
|  | Romanchuk 2002  Conner-Spady 2005 | 1. Best-corrected visual acuity in the non-surgery 2. Best-corrected visual acuity in the surgery eye 3. Glare 4. Age-related macular degeneration and other ocular co-morbidity 5. Impairment in visual function 6. Other substantial disability 7. Ability to work, live independently, or care for dependants |
|  | Lundstrom 2006  Ng 2014 | 1. Visual acuity, surgery eye 2. Visual acuity, fellow eye 3. Patient’s perceived difficulty in performing day-to-day activities (assessed by the Priquest questionnaire) 4. Cataract symptoms (glare, difference between the eyes) 5. Ability to live independently (work, driving,home help, caring for relatives, etc.) 6. Medical ⁄ ophthalmic reasons for urgent surgery |
|  | Fantini 2004 | 1. Type of surgery (unilateral or bilateral, with or without coexisting disease) 2. Visual acuity (in the operated eye and in the contralateral eye) 3. Visual function 4. Life expectancy 5. Patient's ability to work or live independently. |
| **Other elective surgery** | Dennett 1998  Derrett 2003  McLoead 2004  Dew 2005 | 1. Suffering 2. Disability 3. Clinical cost of delay 4. Degree of improvement anticipated 5. Likelihood of improvement |
|  | Solans 2013 | 1. Disease severity 2. Pain (or other main symptoms) 3. Rate of disease progression 4. Difficulty in doing activities of daily life 5. Probability and degree of improvement 6. Being dependent with no caregiver 7. Limitation to care for one’s dependents (if that be the case) 8. Limitations in the ability to work, study or seek for employment |
|  | Taylor 2002 | 1. Usual frequency of painful episodes/suffering 2. How bad is the pain at its worst? 3. Usual intensity of other forms of suffering 4. Degree of impairment in usual activities due to surgical condition, 5. Recent history of major complications or significant examination/test results 6. Life expectancy implications of the condition without the procedure 7. Expected improvement in life expectancy with surgery 8. Situation urgency |
|  | Valente 2009 | 1. Presence of fast disease progression 2. Grade of pain, dysfunction or disability |
| **Orthodontic treatment** | Brook 1989 | 1. Functional and dental health indications for treatment 2. Aesthetic impairment. |
|  | Mohlin 2003 | 1. Treatment need: based on Swedish Medical Board index, Indication index (low, high, very high), IOTN-Dental Health Component, IOTN-Aesthetic Component, 2. Orthodontic treatment outcome: PAR score. 3. Treatment priority |
| **Psychiatry** | Isojoki 2008 | 1. Danger to self 2. Danger to others 3. Psychotic symptoms 4. Delayed adolescent development 5. Internalizing symptoms 6. Externalizing symptoms 7. Problems at school 8. Problems in peer or social relationships 9. Problems at home 10. General functioning (Children’s Global Assessment Scale score) 11. Problems in the family’s capacity to provide the adolescent with support 12. Somatic comorbidity 13. Psychiatric comorbidity 14. Harmful substance use/misuse 15. Anticipated prognosis without psychiatric treatment |
|  | Kaukonen 2010 | 1. Serious symptoms 2. Internalised symptoms 3. Externalised/disruptive behaviour problems 4. Developmental progress delay/risk of delay 5. Patient’s problems in the context of home 6. School and/or work problems 7. Social/friendships/community functioning problems 8. General functioning of youths (Children’s Global Assessment Scale score) 9. Comorbid medical conditions 10. Comorbid psychiatric conditions 11. Problems in family functioning or factors affecting child 12. Family history of mental illness 13. Harmful substance use/misuse problems 14. Prognosis without further intervention 15. Degree of likely benefit from further intervention |
| **Mental health** | Cawthorpe 2007 | 1. Danger to self 2. Danger to others 3. Psychotic Symptoms 4. Global age-appropriate developmental progress 5. Children’s general functioning (Children’s Global Assessment Scale score) 6. Internalized Symptoms 7. Externalized Symptoms 8. Co-morbid medical conditions 9. Co-morbid psychiatric conditions 10. Harmful substance use/misuse 11. Significant biological family history of mental illness 12. School and/or work 13. Social/friendships/community functioning 14. Does the patient have problems in the context of the home? 15. Family functioning or factors affecting child 16. Prognosis without further intervention 17. Degree of likely benefit with further intervention. |
|  | Boucher 2016 | 1. Symptoms intensity and frequency 2. Decrease in normal functioning 3. Impacts on everyday activities 4. Degree of suffering and clinical complexity 5. (adding Social stress for adult version) 6. Danger to self 7. Degree of likely benefit from immediate intervention   Risk of deterioration without further intervention |
| **Bariatric surgery** | Pérez 2018 | 1. Time on surgical waiting list 2. Body mass index 3. Obesity-related comorbidities 4. Functional limitations. |
| **Chronic care** | Burkell 1996 | 1. Communication problems 2. Swallowing difficulties 3. Skin breakdown 4. Incontinence 5. Cognitive difficulties 6. Behavioural problems 7. Rehabilitative services in use 8. Polypharmacy indications 9. Current placement 10. ADL dependence 11. Mobility limitations 12. Caregiver strains 13. High level of care |
| **Coronary artery bypass surgery** | Seddon 1999. | 1. Degree of coronary artery obstruction 2. Angina: Canadian Cardiovascular Society class after appropriate treatment 3. Exercise stress test (Bruce protocol) 4. Left ventricular function 5. Ability to work, care for dependents or live independently 6. Age adjustment if >70. |
| **Magnetic resonance imaging** | Hadorn 2002 | 1. Usual duration, frequency, intensity of pain and/or suffering 2. Severity of illness, impairment 3. Probability of MRI providing clinically significant diagnostic information 4. Probable time course of clinical deterioration 5. Probability of successful treatment resulting from the diagnostic information |
| **Occupational therapy** | Heasman 2012 | 1. Overactive, aggressive, disruptive or agitated behaviour 2. Non-accidental self-injury 3. Problem-drinking or drug-taking 4. Cognitive problems 5. Physical illness or disability problems 6. Problems associated with hallucinations and delusions 7. Problems with depressed mood 8. Other mental and behavioural problems 9. Problems with relationships 10. Problems with activities of daily living 11. Problems with living conditions 12. Problems with occupation and activities |
| **Physiotherapy** | Mifflin 2010 | 1. Hospital inpatients 2. Paediatric clients with chronic conditions and disabilities 3. Clients at risk for falls 4. Clients with urgent orthopaedic conditions 5. Adult clients with chronic conditions and disabilities 6. Clients followed by home care – non-urgent issues 7. Adult clients with non-urgent orthopaedic conditions. |
| **Psychotherapeutic service** | Walton 2002 | Suicidality:   1. Present stress 2. Symptoms 3. Thoughts of suicide 4. Current plan 5. Prior suicidal behaviours 6. Available supportive resources. 7. Severity of Presenting Problem. 8. Strength of Internal Coping Resources 9. Availability of Interim Care Options.   Possible Negative Impact of Waiting:   1. Significant deterioration of mental health 2. Loss of openness to psychological intervention 3. Risk of self harm/harm to others 4. Previous history of difficulty accessing services 5. Anniversary reactions as an aspect of presentation. |
| **Rheumatology** | Fitzgerald 2011 | 1. Self-reliance / Independence 2. Limit to usual work/role 3. Pain 4. Currently receiving corticosteroids for the referred condition 5. Complexity of management due to comorbidities 6. Evidence of progressive major organ involvement 7. Presence of inflammatory markers 8. Evidence of active inflammatory arthritis |
| **Varicose vein surgery** | Montoya 2014 | 1. Clinical manifestation 2. Varicose vein size 3. Presence of complications 4. Influence on quality of life (CIVIQ score) 5. Presence/absence of aggravating work conditions |
| **Elective admission** | Zhu 2019 | 1. Severity level (urgency) 2. Disease type (disease complexity) 3. Waiting time (duration from bed application to the decision epoch) 4. Dual referral (whether transferred from lower-level hospitals) 5. Insurance type 6. Insurance location (efficiency driven performance metric) 7. Case mix index (referred to as disease complexity) 8. Research value (referred to as contribution to clinical research) 9. Diagnosis-related groups cost (referred to as total treatment charge for a single type of disease) 10. Average length of stay (referred to as expected treatment duration from admission to disposition). |
