# Supplementary material for: A systematic review of patient prioritization tools in non-emergency healthcare services
Source: Syst Rev. 2020 Oct 6;9:227. doi: 10.1186/s13643-020-01482-8 (PMC7541289; doi:10.1186/s13643-020-01482-8)
Supplement: Supplementary file 4 — Additional file 4. Key results regarding reliability and validity of PPTs. [file 13643_2020_1482_MOESM4_ESM.docx]

## Additional file 4

## Key results regarding reliability and validity of PPTs

| **Healthcare service** | **Reference** | **Reliability** | **Validity** |
| --- | --- | --- | --- |
| **Arthroplasty** | Allepuz 2008 | The inter-observer ICC was 0.79 (CI 95%: 0.64–0.94), **acceptable**. | Correlation of the overall priority score with the doctor's opinion on surgical priority (VAS) was **strong** (0.64, CI 95%: 0.60–0.68)  Correlation of the patient's perception of the difficulty caused by the condition was **moderate** (0.31, CI 95%: 0.24–0.38).  Correlations were **moderate** with the WOMAC (0.39, CI 95%: 0.33–0.45).  Correlation was **low** with the HUI3 (0.23, CI 95%: 0.11–0.36) and it was **moderate** with the EQ-5D (0.36, CI 95%: 0.26–0.45). |
|  | Escobar 2007  Escobar 2009 | Agreement among panelists reached 35.4% in the first round and 42.2% in the second round. The level of disagreement was very low (1%). The intra-class correlation coefficient for the first round was 0.56 and for the second 0.72. Inter-rater reliability was **good**. | Both face validity and the professionals’ and patients’ viewpoints were considered, as the variables and their levels were chosen by a panel of experts used to develop the RAND method and focus groups developed with patients on waiting lists.  The correlation between the priority score and the WOMAC questionnaire dimensions were 0.79 (function), 0.69 (pain) and 0.51(stiffness). These figures were similar in the knee joint (0.78, 0.69, 0.50) and the hip joint (0.76, 0.70, 0.51). All of them were statistically significant. The global convergent validity, measuring similar construct, ranged from 0.45 to 0.68. **Moderate to good** validity. |
|  | Quintana 2000 | Intra-rater: a weighted kappa of the main panel was 0.81 (95% CI, 0.68, 0.95).  Inter-rater: the second panel compared with the main panel gave a weighted kappa test of 0.77 (95% CI, 0.71, 0.83). A comparison of main panel (panel 1) results with the evaluations made by those 107 orthopedic surgeons indicated high agreement with the main panel, measured by a weighted kappa of 0.89 (95% CI, 0.8, 0.99). **Good** reliability. | The physical and pain domains of the SF-36 showed important improvements in those indications considered appropriate and uncertain. This was also reflected by the three dimensions of the WOMAC questionnaire. In contrast, those indications considered inappropriate showed a 12-point improvement in physical function and 3.5-point improvement in pain on the SF-36 questionnaire, and an 18-point improvement in pain, and a 14-point improvement in the functional limitations ares of the WOMAC questionnaire. **Acceptable** validity. |
|  | Arnett 2003  Conner-Spady 2004  Conner-Spady 2004 | One of the 7 criteria items had **excellent** reliability (ICC > 0.75); 5 had **fair to good** reliability; and 1 (potential for progression of disease) had **poor** reliability. Relatively high intra-rater consistency in scoring was observed over the 5- to 6-month interval; 3 criteria had ICC values in the excellent range and 3 in the fair to good. Reliability was also studied with 11 general practitioners as raters (Table 3). ICC values were quite comparable to those for orthopedic surgeons as well as raters from related clinical fields. **Good** inter-rater agreement and **good** intra-rater stability.  Internal consistency: Coefficient alpha was .79. **Acceptable** reliability. | The multiple correlation (R) between the combined 7 priority criteria and the VAS urgency was 0.82, whereas the correlation of the PCS with the 5-point relative urgency scale was 0.78. Correlations between the PCS and the WOMAC function subscale were 0.48. Convergent validity was **moderate**, ranging from 0.45 to 0.56.  Correlations between the PCS and VAS urgency rating were **high** (0.89), whereas MAWTs were **moderately** correlated with the PCS (–0.67).  Correlations between the surgeon-rated measures ranged from 0.79 (PCS and VAS urgency) to 0.38 (MAWT with PCS and VAS urgency). Correlations between the PCS and patient-rated measures were 0.26 (EQ-VAS), 0.33 (EQ-5D), 0.33 (WOMAC), and 0.38 (patient MAWT).  Convergent validity coefficients (those measuring similar constructs) ranged from 0.24 to 0.32. The correlation between surgeon rated pain at rest and patient-rated pain at night (0.32) and pain sitting or lying (0.25) were higher than dissimilar constructs, for example, surgeon-rated pain at rest and patient-rated walking (0.20) (discriminant validity). Surgeon-rated functional limitation (Question 4) was more highly related to WOMAC ascending and descending stairs (0.26) and function (0.28) than to WOMAC pain at night (0.15) and pain sitting or lying (0.08) (discriminant validity). **Weak to moderate** validity. |
|  | De Coster 2007 | The ICC was 0.78 for all reviewers, 0.76 for primary care providers and 0.87 for orthopaedic surgeons. The PRS had **excellent** inter- and intra-rater reliability and was The ICC coefficient for the first phase was 0.73; the test–retest ICC was 0.84. The ICC for each of the raters was calculated and the mean intra-rater ICC was 0.79; 15 of the individual ICCs were 0.75 or more | Seen to have face validity by the Primary Care Panel. |
|  | Coleman 2005 |  | The Pearson correlation coefficient was 0.261 when comparing the priority scoring system with the WOMAC score, and 0.194 when comparing to the MFA indicating **poor** correlation of the scoring systems. |
|  | Theis 2004 |  | The priority scores used in GPSS correlate **well** with the quality of life of patients in the sense that those patients who are selected and booked for surgery have worse quality of life as compared to those not qualifying for surgery. |
| **Cataract surgery** | Allepuz 2008  Comas 2008 | The inter-observer ICC was 0.79 (0.79, CI 95%, 0.63–0.95), **acceptable**. | The correlation of the overall priority score with the doctor's opinion on surgical priority (VAS) was **strong** (0.65, CI95%: 0.61–0.69) The correlation of the patient's perception of the difficulty caused by the condition was **moderate** (0.31, CI95%: 0.26–0.36).  Face validity: The panel of experts considered all results as valid and credible and the model as useful in achieving the established objectives. |
|  | Gutierrez 2009 |  | High-priority patients experienced greater improvement in VA and HRQoL than those classified as intermediate or low-priority. Interestingly, the time patients spent on waiting lists was similar for patients regardless of priority classification.  Face validity: The variables that composed the clinical scenarios used in the process were chosen by a panel of ophthalmologists based on their clinical judgment. In the high-priority group, the benefits declined as the waiting time increased (predictive validity). |
|  | Romanchuk 2002  Conner-Spady 2005 | There was **poor** interrater agreement on the ratings of urgency for the six standardized patients (ICC 0.25); agreement was somewhat **higher** for ophthalmologists (ICC 0.44) (Table 3).  There was **excellent** interrater agreement (ICC greater than 0.75) for four criteria items, **fair to good** agreement for one item, and **poor** agreement (ICC less than 0.40) for three items. | The correlation between VF-14 scores and ratings on the WCWL global item of visual functional impairment (item 4) was **high** (correlation coefficient 0.71).  Correlation between the PCS and physician-rated VAS Urgency was 0.65, **moderately strong**  Correlations between the PCS and pre-surgery VFA (−0.35) were **low**. |
|  | Lundstrom 2006 | The test-retest reliability test of the indication grouping showed an intraclass correlation coefficient (ICC) of 0.526 (95% confidence interval 0.323–0.682, p < 0.001) and the examiner variation test of the indication grouping showed an ICC of 0.923 (95% CI 0.878–0.952, p < 0.001). **Good** reliability. | There was no statistically significant correlation between the scores achieved by the two methods, but neither did they differ very much from one another (ICC = 0.275; 95% CI - 0.231 to 0.573, p = 0.116). The reduction in each item area was greatest in IG 1. In IG 4 some item areas (perceived difficulties in day-to-day life and cataract symptoms) even deteriorated after surgery. **Good** validity. |
| **Other elective surgery** | Dennett 1998  Derrett 2003 |  | CC for all data was 0,46 (p<0,0001). **Poor** correlation between the LAS and GSPC and the limits of agreement analysis show no agreement.  Ranking of the CPAC scores for all patients was **weakly** related to the ranking of their EQ-5D values (r=−0.34). The latter were more closely correlated with the PCS (r=0.60) and the combined condition related (i.e., VF-14, IPSS, and ISH/ISK) scores (r=0.51), and were also **weakly** correlated in an unexpected direction with the MCS (r=−0.19). The CPAC and condition-related scores were **strongly** correlated (r=−0.60); however, there was only a **weak** correlation with the PCS (r=−0.25), and **no significant** relationship with MCS. |
|  | Taylor 2002 | Results of the reliability assessment of the revised priority criteria (Table 3) found **excellent** interrater agreement for the VAS urgency ratings for the 6 standardized patients (ICC = 0.83). Three criteria had **excellent** reliability (ICC > 0.75); 3 items had **fair to good** reliability and 1 item had **poor** reliability (ICC < 0.40).  Relatively **good** intrarater consistency in scoring was found for the majority of criteria items. |  |
| **Orthodontic treatment** | Brook 1989 | Intra-examiner agreement ranged from a Kappa value of 0.837 for the referred population seen under ideal conditions, to 0.754 for the nonreferred population.  Inter-examiner agreement ranged from 0.731-0.797. In total there were 21 out of 154 measurements that were not agreed. There were only 2 cases where the error was by more than one grade. |  |
| **Psychiatry** | Kaukonen 2010 | Interrater reliability between the SMC level raters for each of the individual 2–4-level items was **moderately good**; kappa was at least 0.40 for 10 items and 0.22–0.36 for the others. The ICC value for the continuous clinical necessity-for-treatment assessment was 0.65 and kappa for the dichotomised version 0.59. The ICC value for the Selected Finnish Sum Score was 0.66 and kappa 0.41. | The validity of the Finnish Criteria Tool was confirmed by the results among the non-urgent SMC group (N= 244). Most of the individual items correlated at least moderately well with the clinical necessity-for-treatment assessment; the correlation was above 0.45 for the serious symptoms item, CGAS and prognosis without treatment, and below 0.30 for somatic comorbidity, harmful substance use, problems in family functioning, family history of mental illness, and degree of likely benefit with treatment, the rest being in between. The correlation of the score sum with the necessity-for-treatment assessment was 0.66. The sensitivity and specificity of the tool in this group were 80%. |
| **Mental health** | Cawthorpe 2007 | The internal consistency of the PCS items was **excellent** (coefficient alpha = .76) indicating that the items were measuring a similar construct. | The VAS (r = .67), MAWT (r = –.58), and actual wait times (r = –.25) were related significantly with the PCS (p < .05). |
|  | Boucher 2016 | Results of **strong** reliability for RIFCA scores, ICC = 86, p < 0,05  Interrater agreement is **strong** between total priority score and all factors (from r = 0,98 to r = 0,92), except for functioning foactor (r = 0,78). | **Strong** relation between priority determined by “guichets” and tool scores, r = 0,57. **Weaker** relation between priority scores avec evaluation by child psychiatrist, r = 0,45.  Result of correlation between the priority tool and the prior usual tool is r = 0,67 (n = 120). |
| **Magnetic resonance imaging** | Hadorn 2002 | There was **poor** inter-rater agreement among the 7 raters (neurologist and neurosurgeons) who reviewed the paper cases in January 2001 for the VAS urgency ratings for the 6 patients (ICC = 0.38). There was **fair to good** agreement for item 1 (ICC =: 0.60) and **poor** agreement (ICC < 0.40) for the remaining 4 items. Test-retest (intra-rater) reliability, measured over a 2-month interval, appeared **stronger**, with 1 item in the **excellent** range (ICC > 0.75) and the remaining items having **fair to good** intra-rater agreement (ICC - 0.50-0.75). |  |
| **Psychotherapeutic service** | Walton 2002 | The inter-rater reliability results suggest that the clinicians consistently made triage ratings of the vignettes in a similar manner. Internal Overall alpha coefficient of reliability for the CPRS = 0.77 | Face validity of the scale items is supported in part by the similarity of the suicidality items in the CPRS to the Dangerousness item in the Crisis Triage Rating Scale a well-validated scale (see Turner and Turner, 1991) scores on treatment variables. The finding of a **moderate** relationship between CPRS score and GAF supports the view that a component of the CPRS is related to severity and serves as a measure of validity. |
| **Rheumatology** | Fitzgerald 2011 | With initial testing, the interrater ICCs for the PRS were 0.80 and 0.81 for the rheumatologists and PCPs, respectively. With retesting, the average intra-rater ICC for the PRS was 0.83 for the rheumatologists and 0.82 for the PCPs |  |
| **Varicose vein surgery** | Montoya 2014 | A total of six dominance factors were detected between cards and for all the pairs approximately 80% of the participants put every dominant factor in the correct order. When comparing all dominant factors, concordance reached 70,2% and this value guarantees the **consistency** of the prioritization performed by participants. | We observed that the coefficients for the parameters increased as the characteristics of the hypothetical patient worsened. Spearman's correlation coefficient values were **high** (r = 0,98 p<0,001). Importance given by each participant to each of the 5 factors to determine priority order (clinical manifestation, complications, quality of life) coincided with the order of importance shown using card. Relative importance of the 2 other factors was not in agreement with the relative importance obtained with the cards. |

CGAS = Children’s Global Assessment Scale, CI = confidence interval, CPAC = clinical priority assessment criteria, CPRS = Client Priority Rating Scale, GAF = Global Assessment Functioning, GPSS = Generic Priority Scoring System, GSPC = Generic Surgical Priority Criteria, HRQoL = Health Related Quality of Life, HUI3 = Health Utilities Index Mark 3, ICC = intraclass correlation coefficient, IPSS = International Prostate Symptom Score, ISH/ISK = Lequesne Indices of Hip/Knee Severity, LAS = Linear Analogue Scale, MAWT = maximum acceptable waiting times, MCS = Mental Component Summary Score, MFA = Musculoskeletal Function Assessment, PCP = primary care providers, PCS = priority criteria score, PRS = Priority Referral Score

SMC = specialised medical care, VA = visual acuity, VAS = visual analogue scale, VF-14 = Visual Function-14, VFA = Visual Function Assessment, WCWL = Western Canada Waiting List, WOMAC = Western Ontario and McMaster Universities Osteoarthritis Index
